# Treadmill Exercise Facilitates Synaptic Plasticity in *APP/PS1* Mice by Regulating Hippocampal AMPAR Activity

**DOI:** 10.3390/cells13191608

**Published:** 2024-09-25

**Authors:** Laikang Yu, Yan Li, Yuanyuan Lv, Boya Gu, Jiajia Cai, Qing-Song Liu, Li Zhao

**Affiliations:** 1 Department of Strength and Conditioning Assessment and Monitoring, Beijing Sport University, Beijing 100084, China; yulaikang@126.com; 2 Beijing Key Laboratory of Sports Performance and Skill Assessment, Beijing Sport University, Beijing 100084, China; bsuliyan@bsu.edu.cn (Y.L.); sunflowerlyy@bsu.edu.cn (Y.L.); guboya@bsu.edu.cn (B.G.); caijj_2008@163.com (J.C.); 3 China Institute of Sport and Health Science, Beijing Sport University, Beijing 100084, China; 4 Department of Pharmacology and Toxicology, Medical College of Wisconsin, Milwaukee, WI 53226, USA; qsliu@mcw.edu

**Keywords:** exercise, synaptic plasticity, AMPA receptor, Alzheimer’s disease

## Abstract

Accumulating evidence underscores exercise as a straightforward and cost-effective lifestyle intervention capable of mitigating the risk and slowing the emergence and progression of Alzheimer’s disease (AD). However, the intricate cellular and molecular mechanisms mediating these exercise-induced benefits in AD remain elusive. The present study delved into the impact of treadmill exercise on memory retrieval performance, hippocampal synaptic plasticity, synaptic morphology, and the expression and activity of α-amino-3-hydroxy-5-methyl-4-isoxazolepropionic receptors (AMPARs) in 6-month-old *APP/PS1* mice. *APP/PS1* mice (4-month-old males) were randomly assigned to either a treadmill exercise group or a sedentary group, with C57BL/6J mice (4-month-old males) as the control group (both exercise and sedentary). The exercise regimen spanned 8 weeks. Our findings revealed that 8-week treadmill exercise reversed memory retrieval impairment in step-down fear conditioning in 6-month-old *APP/PS1* mice. Additionally, treadmill exercise enhanced basic synaptic strength, short-term potentiation (STP), and long-term potentiation (LTP) of the hippocampus in these mice. Moreover, treadmill exercise correlated with an augmentation in synapse numbers, refinement of synaptic structures, and heightened expression and activity of AMPARs. Our findings suggest that treadmill exercise improves behavioral performance and facilitates synaptic transmission by increasing structural synaptic plasticity and the activity of AMPARs in the hippocampus of 6-month-old *APP/PS1* mice, which is involved in pre- and postsynaptic processes.

## 1. Introduction

Alzheimer’s disease (AD), a prominent neurodegenerative disease, is marked by a gradual deterioration in cognitive functions and memory capabilities [1]. Its incidence has escalated significantly, transforming from affecting approximately 3% of individuals aged 65–74 to nearly half of those over 85 years old [2]. Alarmingly, forecasts predict that the global population of AD patients will surpass the 100 million mark by 2050, underscoring the urgency of addressing this issue [3].

Synaptic dysfunction is a major contributor to cognitive deficits in the initial stages of AD [4]. Complex synaptic compensatory mechanisms, including enhanced synaptic transmission and synaptic connectivity, work to maintain normal cognitive performance. Notably, the hippocampus emerges as the primary target of damage in the *APP/PS1* mouse strain, where intracellular β-amyloid peptide (Aβ) accumulates at very early stages [5]. Long-term potentiation (LTP), a cellular mechanism of learning and memory, involves the dynamic recruitment of α-amino-3-hydroxy-5-methyl-4-isoxazolepropionic receptor (AMPAR) at synaptic sites, accompanied by a concurrent increase in AMPA-mediated transmission [6,7].

Previous studies have revealed impairments in LTP across different ages of *APP/PS1* mice. For instance, Krishna-K et al. [8] observed that LTP is attenuated in individual pyramidal neurons of 3-month-old *APP/PS1* mice. Additionally, He et al. [9] demonstrated that 6-month-old *APP/PS1* mice exhibited long-term spatial memory impairment and in vivo hippocampal LTP suppression compared to wild-type mice. Furthermore, Liu et al. [10] showed that 8-month-old *APP/PS1* mice, when compared to wild-type mice, were characterized by LTP deficits. Moreover, Gruart et al. [11] reported a significantly lower LTP in 12-month-old *APP/PS1* mice compared to that of wild-type mice.

In mature hippocampal CA1 neurons, AMPARs are mainly composed of complex isoforms of the GluA2 subunit and the GluA1/3 subunit [12]. At most excitatory synapses in the brain, AMPARs express the GluA2 subunit, rendering the channels Ca^2+^-impermeable or hypo-permeable [13]. Ca^2+^ influx through NMDA receptors triggers LTP. Regulation of GluA2 expression/activity can have a substantial impact on synaptic function and neuronal survival [14]. AMPAR-mediated neuronal activity exerts profound influences on multiple aspects of synaptic organization and function, including excitatory synapse establishment and stabilization, synaptic efficacy, plasticity, and neural circuit architecture. During LTP induction, a pivotal event involves the phosphorylation of GluA1 carboxy-terminal (C-tail) residue S831 by calcium/calmodulin-dependent protein kinase II (CaMKII) and protein kinase C (PKC). This phosphorylation enhances AMPAR’s open probability and single-channel conductance [15,16]. Additionally, LTP triggers the phosphorylation of GluA1 S845 by protein kinase A (PKA), which is crucial for directing GluA1 to the postsynaptic membrane. This process, known as “priming”, prepares AMPARs for synaptic trafficking and incorporation, further modulating synaptic plasticity [17,18]. Various mechanisms can alter AMPAR composition at synaptic sites. For example, prolonged synaptic inactivation in hippocampal neurons increases local translation of GluA2-lacking AMPARs [19], while ischemic insults silence GluA2 expression in selectively vulnerable CA1 neurons by inducing transcriptional repressors [20]. Consequently, Ca^2+^-permeable AMPARs assume a pivotal role in neurodegenerative diseases associated with neuronal death. Many neurodegenerative diseases are directly related to changes in AMPAR synaptic signaling [15,21]. Reduced surface expression of the AMPAR complex likely underlies certain biochemical, electrophysiological, and behavioral impairments characteristic of AD [22,23]. However, a previous study reported that male AD patients exhibited a notable increase in GluA1 in cortical tissues at 3 h postmortem, whereas the receptor complex consisting of GluA2-4 remained unchanged [24]. These findings indicate that alterations in receptor complex levels may vary depending on the preclinical or clinical AD stage, leading to a deterioration in the synergistic activity of these receptors.

Globally, despite concerted efforts, an effective treatment for AD remains elusive, necessitating the urgent pursuit of preventative strategies to mitigate its early progression. Emerging epidemiological research underscores exercise as a feasible and cost-effective lifestyle intervention that can diminish AD risk and postpone its manifestation and advancement [25,26,27]. Data from different transgenic lines of AD mice suggest that exercise can prevent cognitive decline or protect brain function by directly affecting Aβ or the hyperphosphorylation of protein tau [28,29,30], which are major players in AD pathology. The impact of exercise on the glutamatergic system, particularly its modulation of synaptic AMPAR composition, is contingent upon the exercise’s type, duration, and intensity [31,32,33,34].

Given that AD’s pathological processes may initiate decades before symptomatic onset [35,36], early exercise intervention holds promise in delaying the emergence of AD symptoms. Therefore, this study delved into the consequences of treadmill exercise on memory retrieval and hippocampal synaptic plasticity and morphology in *APP/PS1* mice. The examination of the mice was conducted at the age of 4 months, preceding the emergence of Aβ deposition, a phenomenon that typically manifests around the 6th month of their lifespan [37]. Our aim was to determine whether exercised-enhanced synaptic plasticity is correlated with alternations in AMPARs.

## 2. Materials and Methods

### 2.1. Animals

Thirty male 4-month-old *APP/PS1* (Tg) mice and an equal number of C57BL/6J (Wt) mice were procured from Beijing HFK Bioscience Co., Ltd. (Beijing, China), adhering to the ethical guidelines approved by the ethics committee of Beijing Sport University.

### 2.2. Exercise Training Protocol

**Figure 1:**
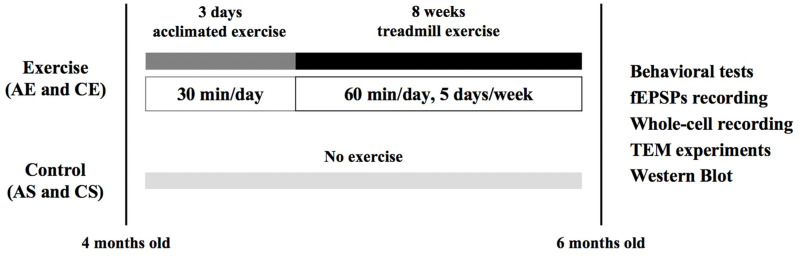
Experimental design. Mice destined for exercise underwent a 3-day treadmill acclimation period. Over an 8-week period, the exercise groups ran daily for 60 min, 5 days per week. Behavioral performance and molecular changes were assessed after the intervention. AS, *APP/PS1* sedentary group; AE, *APP/PS1* exercise group; CS, C57BL/6J sedentary group; CE, C57BL/6J exercise group.

The mice were stratified randomly into four groups: *APP/PS1* sedentary group (AS, *n* = 15), *APP/PS1* exercise group (AE, *n* = 15), C57BL/6J sedentary group (CS, *n* = 15), and C57BL/6J exercise group (CE, *n* = 15). As shown in Figure 1, all mice destined for exercise underwent a 3-day acclimation period on the treadmill, with the first day at 5 m/min and subsequent days at 10 m/min for 30 min each. For an 8-week period, the exercise groups engaged in daily treadmill exercise for 60 min, 5 days a week, with a gradual speed increase from 12 m/min or the initial 10 min to 15 m/min for the remaining 50 min, all at 0% incline [38]. Meanwhile, the sedentary groups remained on the treadmill for an equivalent period without engaging in running.

### 2.3. Behavior

Behavioral changes were detected by step-down inhibitory avoidance task. Individual mice were subsequently positioned on a cylindrical pedestal (r = 4 cm, h = 5 cm) that was placed on a metal grid inside an experimental box (14 × 13 × 43 cm). Upon fully stepping off the pedestal onto the lattice with all four limbs, the mice underwent a 28 V foot shock, which was the minimum voltage that could cause the mouse to escape. The mice were then returned to their cages, and the platform and experimental box were cleaned before the next mouse was tested. After an interval of one hour, a repeat test was administered, during which the time taken to step down was noted as the primary latency measure. Subsequently, 24 h post the initial testing phase, the mice were reinstated on the pedestal for a second evaluation, with the latency recorded on this occasion serving as the secondary latency indicator. During this test, the frequency of stepping off the platform or remaining on the platform for 5 min was also noted. Mice whose second latency was at least three times longer than their first latency were considered to have met the avoidance criterion. No foot shocks were administered during the second test.

### 2.4. Transmission Electron Microscope (TEM)

To prepare the TEM samples, the excised hippocampal CA1 region was promptly sectioned into minute fragments, each no larger than 2 mm^3^, and promptly immersed in 2.5% glutaraldehyde at 4 °C for an overnight period. The following day, the tissue fragments underwent a series of four rinses with 0.1 M PB, each lasting 15 min, followed by a 2 h fixation with 1% osmium tetroxide. The tissue pieces were then dehydrated with gradient acetone at concentrations of 30%, 50%, 70%, 80%, 90%, 100%, 100%, and 100% for 15 min each. Following dehydration, the tissue fragments were infiltrated with an acetone–resin blend in sequential ratios of 1:1, 1:2, and 1:3 for durations of 1.5 h, 1.5 h, and 2 h, respectively. Afterwards, they were embedded in the resin overnight, and the tissue pieces were cut into ultrathin sections (60 nm) on a Reichert Ultra cut microtone, mounted on 300-mesh grids (TAAB Laboratories Equipment Ltd, Aldermaston, England, UK), subjected to staining with uranyl acetate and lead citrate, and finally rinsed with water.

Ultrathin sections were observed on a JEOL JEM-2100F transmission electron microscope (Tokyo, Japan) at ×30,000 magnification, and 5 samples were selected from each group, with 8 randomly selected visual fields for each sample, of which photographs were taken. Electron micrographs were captured by two unbiased observers, unaware of the sample group allocations. Likewise, each micrograph was assessed by two independent observers who were blind to the group identity of the images. Synapse counts adhered to specific criteria: firstly, a minimum of three synaptic vesicles present in the presynaptic neuron; secondly, a clearly discernible postsynaptic density (PSD) in the postsynaptic neuron, as per established guidelines [39]. Furthermore, we measured the synaptic interface curvature, the length of the synaptic active zone, thickness of the PSD, and the width of synaptic cleft, utilizing protocols outlined in our previous research [38].

### 2.5. Electrophysiology

#### 2.5.1. Brain Slice Preparation

Animals were anesthetized with isoflurane and underwent transcardial perfusion with an N-Methyl-D-glucamine (NMDG) solution tailored to maintain a pH range of 7.2–7.4 (adjusted with HCl). This solution comprised various components, including 92 mm NMDG, 2.5 mm KCl, 1.25 mm NaH_2_PO_4_; 30 mm NaHCO_3_; 20 mm HEPES; 25 mm glucose; 2 mm Thiourea; 5 mm Na ascorbate; 3 mm Na pyruvate; 0.5 mm CaCl_2_·2H_2_O; and 10 mm MgSO_4_·7H_2_O. Subsequently, the entire brain was excised and promptly immersed in chilled NMDG solution, followed by embedding in 4% low-gelling agarose to facilitate sectioning. Coronal hippocampal slices, each 300 μm thick, were meticulously prepared using a VT1200s vibrating slicer (Leica Microsystems, Wetzlar, Germany).

The prepared brain slices were initially incubated in an oxygenated NMDG solution for 10 min before being transferred to and stored in an oxygenated artificial cerebrospinal fluid (ACSF) solution for at least 1 h, specifically designed to mimic the brain’s physiological environment. This ACSF solution 3 mm KCl, 2 mm CaCl_2_·2H_2_O, 1.3 mm MgCl_2_·6H_2_O, 1.25 mm NaH_2_PO_4_, 119 mm NaCl, 25 mm NaHCO_3_, and 10 mm glucose, ensuring optimal conditions for neuronal function [40]. Both solutions were continuously saturated with 95% O_2_ and 5% CO_2_.

#### 2.5.2. Field Excitatory Postsynaptic Potentials (fEPSPs) Recording

To evoke fEPSPs, we used a bipolar tungsten electrode to stimulate the Schaffer collateral/commissural pathway at a frequency of 0.033 Hz, while field recordings were made using glass pipettes filled with 1 M NaCl and positioned in the stratum radiatum of the CA1 region of the hippocampal slices. The intensity of stimulation was incrementally adjusted to generate input/output (I/O) curves, which were plotted by correlating the fEPSP slope with the presynaptic fiber volley amplitude [41]. Additionally, we recorded paired-pulse facilitation (PPF) at an intensity that elicited approximately 40% of the maximal evoked response, utilizing various interpulse intervals including 20, 50, 100, 200, and 400 ms [42]. For LTP recording, a stable baseline of fEPSPs was established for at least 20 min prior to inducing LTP with theta burst stimulation (TBS). TBS consisted of a series of 15 bursts, each containing 4 pulses delivered at 100 Hz, with a 200 ms inter-burst interval. All recordings were conducted at a tightly controlled temperature of 32 ± 1 °C.

#### 2.5.3. Whole-Cell Recording

Patch electrodes, ranging in resistance from 3 to 5 MΩ, were crafted from 1.5 mm borosilicate glass utilizing the P-97 flaming/brown micropipette puller (Sutter Instruments, Novato, CA, USA). The pipettes were filled with an internal solution comprising 140 mm CsCl, 10 mm HEPES, 1 mm EGTA, 2.5 mm MgATP, 0.3 mm Na_3_GTP, 2 mm MgCl_2_, and 5 mm Tetraethylammonium chloride (TEA-Cl), adjusted to pH 7.2 with CsOH. To facilitate dispersion, the GABAA receptor antagonist picrotoxin (100 μm) was dissolved in ACSF via sonication for approximately 15 min. Following recovery, a single slice was transferred to the recording chamber where it was continuously perfused with ACSF at a rate of 2–4 mL/min. Whole-cell recording was made from the hippocampal pyramidal neurons. The stimulating pipette was placed on the axon of a cell. To assess the AMPAR/N-methyl-d-aspartic acid receptor (NMDAR) ratio (AMPAR/NMDAR), neurons were voltage-clamped at +40 mV, enabling the recording of both fast AMPAR- and NMDAR-mediated excitatory postsynaptic currents (EPSCs). Following a 5-min baseline period, the NMDAR antagonist D-2-amino-5-phosphonovaleric acid (D-AP-5, 50 μM) was introduced into the bath for 6–10 min, effectively isolating AMPAR-mediated EPSCs. By subtracting the AMPAR-mediated component from the total EPSCs recorded in the same cell, NMDAR-mediated EPSCs were derived (peak amplitude) [43]. All recordings were conducted at a tightly controlled temperature of 32 ± 1 °C.

### 2.6. Western Blot

Tissues from different groups were collected and homogenized in ice-cold RIPA lysis buffer (Beyotime Institute of Biotechnology, Nantong, China). Following centrifugation at 4 °C for 30 min at 13,000× *g*, the resulting supernatant was carefully extracted. Protein concentrations were quantitatively assessed employing the BCA assay. Equal amounts (20 μg) of sample were added to adjacent duplicate lanes and separated on SDS-PAGE (configured with a 12% spacer gel and run at 120 V for 50 min). The proteins were then electrophoretically separated and transferred onto PVDF membranes under different conditions: 250 mA for 1 h for CaMKIIα, PKA, and GAPDH; and 250 mA for 4 h for GluA1, GluA1 S831, and GluA1 S845. Prior to antibody probing, the membranes were blocked in 5% BSA for 2 h at 37 °C. Subsequently, they were incubated overnight at 4 °C with primary antibodies specific to GAPDH (1:1000, Proteintech Group, Chicago, IL, USA), GluA1 and its phosphorylated forms GluA1 S831 and S845 (each 1:2000, Abcam, Cambridge, UK), PKA (1:2000, Abcam, Cambridge, UK), and CaMKIIα (1:1000, Sigma-Aldrich, St. Louis, MO, USA). The following day, the membranes underwent rigorous washing with TBST (three times for 10 min each) and were then incubated with corresponding secondary antibodies (GAPDH and CaMKIIα at 1:15,000, Proteintech Group, Chicago, IL, USA; GluA1, GluA1 S831, GluA1 S845, and PKA at 1:20,000, Proteintech Group, Chicago, IL, USA) for 1 h at 37 °C. Protein bands were finally visualized using enhanced chemiluminescence (Thermo Scientific Pierce, Waltham, MA, USA).

### 2.7. Statistical Analysis

For statistical analysis, all data were reported as mean ± SEM. The LTP magnitude (%) was derived by the formula: 100 × (final 10-min mean fEPSPs slope/baseline mean fEPSPs slope). PPF was calculated as the ratio of the second EPSP’s mean amplitude to that of the first. The normality of data obtained was checked for all the variables using the Shapiro–Wilk test. Statistical significance was determined at *p* < 0.05, using the Chi-square test for the standard rate of step-down fear conditioning test and two-way ANOVA with Tukey’s post hoc test. Data analyses were executed with SigmaPlot 11.0 software (Systat Software Inc., San Jose, CA, USA).

## 3. Results

### 3.1. Treadmill Exercise Reversed Memory Retrieval Deficits in 6-Month-Old Tg Mice in a Step-Down Inhibitory Avoidance Task

**Figure 2:**
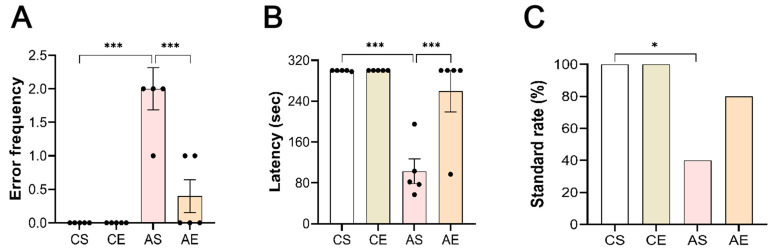
Treadmill exercise reversed memory retrieval deficits in 6-month-old Tg mice on a step-down inhibitory avoidance task. (**A**) Compared with CS, the error frequency was significantly higher (*p* < 0.001); the latency significantly decreased (*p* < 0.001); (**B**) and the standard rate was lower (*p* = 0.038) (**C**) in 6-month-old Tg mice. Eight weeks of aerobic exercise significantly improved the behavioral performance (error frequency, *p* < 0.001; latency, *p* < 0.001) (**A**), (**B**) and increased the standard rate (**C**). AS, *APP/PS1* sedentary group; AE, *APP/PS1* exercise group; CS, C57BL/6J sedentary group; CE, C57BL/6J exercise group. *, *p* < 0.05; ***, *p* < 0.001.

To examine the influence of genotype and exercise on mouse performance in behavioral assays, we employed the step-down inhibitory avoidance paradigm, a fear-motivated learning task. In the step-down inhibitory avoidance task, animals associate the presence of a platform present in a given situation with the shock to the feet when stepping off the platform. Our analysis using a two-way ANOVA, depicted in Figure 2, uncovered substantial impacts of both genotype (significantly altering error frequency, F_(1,19)_ = 36.000, *p* < 0.001; and latency, F_(1,19)_ = 36.854, *p* < 0.001) and exercise (notably affecting error frequency, F_(1,19)_ = 16.000, *p* = 0.001; and latency, F_(1,19)_ = 16.737, *p* < 0.001), along with a significant interaction between these factors (error frequency, F_(1,19)_ = 16.000, *p* = 0.001; latency, F_(1,19)_ = 17.578, *p* < 0.001). Post hoc Tukey’s tests showed that compared with CS, the behavioral performance of 6-month-old Tg mice was impaired significantly (*p* < 0.001; Figure 2A,B), with a lower standard rate (Figure 2C). After 8 weeks of treadmill exercise, the mice performed better (*p* < 0.001) and had a higher standard rate. Therefore, treadmill exercise is an effective intervention to promote behavioral performance in 6-month-old Tg mice.

### 3.2. Treadmill Exercise Facilitated Basal Synaptic Strength and Short-Term Plasticity (STP) of the Hippocampus in 6-Month-Old Tg Mice

**Figure 3:**
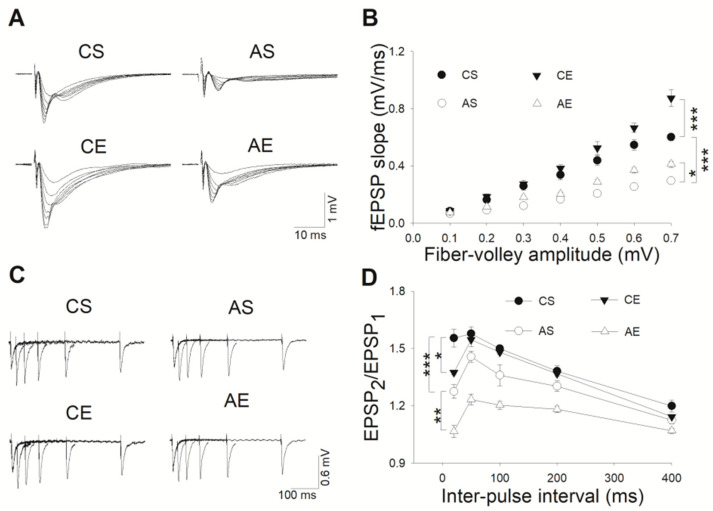
Treadmill exercise facilitated basal synaptic strength and STP within the hippocampus of 6-month-old Tg mice. (**A**) Illustrative recordings of fEPSPs at Schaffer collateral-CA1 synapses, showcasing variations across varying stimulus intensities. (**B**) In comparison to CS, acute hippocampal slice recordings from Tg mice exhibited a marked reduction in AMPA receptor-mediated fEPSPs within the stratum radiatum across all tested stimulation intensities (*p* < 0.001). An eight-week treadmill exercise regimen significantly bolstered the basal synaptic strength in both 6-month-old Tg (*p* = 0.025) and Wt mice (*p* < 0.001). (**C**) Representative traces of paired-pulse EPSPs at interpulse intervals spanning 20–400 ms, encompassing all four experimental groups. (**D**) In contrast to CS, PPF measurements at interpulse intervals of 20–400 ms were significantly diminished in 6-month-old Tg mice (20 ms, *p* < 0.001; 50 ms, *p* = 0.018; 100 ms, *p* = 0.006; 200 ms, *p* = 0.018; 400 ms, *p* = 0.013). Furthermore, eight weeks of treadmill exercise led to a significant decrease in PPF in both 6-month-old Tg mice across the same intervals (20 ms, *p* = 0.004; 50 ms, *p* < 0.001; 100 ms, *p* = 0.002; 200 ms, *p* < 0.001; 400 ms, *p* = 0.047) and Wt mice at 20 ms (*p* = 0.015). AS, *APP/PS1* sedentary group; AE, *APP/PS1* exercise group; CS, C57BL/6J sedentary group; CE, C57BL/6J exercise group. *, *p* < 0.05; **, *p* < 0.01; ***, *p* < 0.001.

Prompted by the cognitive impairments observed in AD, we delved into exploring whether hippocampal synaptic communication and long-term plasticity in 6-month-old *APP/PS1* mice were altered in the disease’s early stages. First, we determined whether basal synaptic strength was affected. As evidenced in Figure 3, our two-way ANOVA analysis disclosed marked effects of genotype (F_(1,23)_ = 129.929, *p* < 0.001) and exercise (F_(1,23)_ = 33.408, *p* < 0.001) on this parameter, alongside a significant genotype–exercise interaction (F_(1,23)_ = 5.471, *p* = 0.030). Post hoc Tukey’s tests highlighted a significant reduction in AMPAR-mediated fEPSPs within the stratum radiatum of acute hippocampal slices from Tg mice, across all tested stimulation intensities, compared that from CS (*p* < 0.001, Figure 3A,B).

PPF is a form of presynaptic Ca^2+^-dependent STP that reflects the probability of neurotransmitter vesicle release; alternations in PPF indicate changes in presynaptic release probability. A two-way ANOVA analysis revealed significant individual contributions of genotype (F_(1,26)_ = 38.430, *p* < 0.001) and exercise (F_(1,26)_ = 16.789, *p* < 0.001) to PPF variations, yet no notable interaction was observed between these two factors (F_(1,26)_ = 0.094, *p* = 0.762). When compared to CS, PPF values at various interpulse intervals ranging from 20 to 400 ms were significantly lower in 6-month-old Tg mice (20 ms, *p* < 0.001; 50 ms, *p* = 0.018; 100 ms, *p* = 0.006; 200 ms, *p* = 0.018; 400 ms, *p* = 0.013, Figure 3C,D). Additionally, treadmill exercise was found to significantly diminish PPF in both 6-month-old Tg mice across all tested intervals (20 ms, *p* = 0.004; 50 ms, *p* < 0.001; 100 ms, *p* = 0.002; 200 ms, *p* < 0.001; 400 ms, *p* = 0.047, Figure 3C,D) and Wt mice, specifically at 20 ms (*p* = 0.015, Figure 3C,D).

### 3.3. Treadmill Exercise Facilitated LTP of the Hippocampus in 6-Month-Old Tg Mice

**Figure 4:**
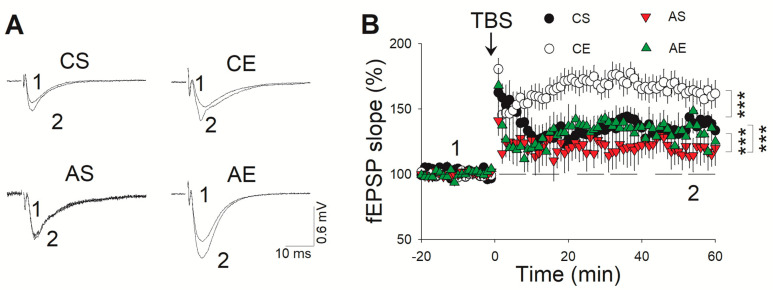
Treadmill exercise facilitated LTP in the hippocampus of 6-month-old Tg mice. (**A**) Representative LTP traces from different groups. (**B**) A markedly diminished fEPSP slope in Tg mice compared to that in Wt mice (*p* < 0.001). An eight-week treadmill exercise regimen robustly augmented the fEPSP slope in both Tg (*p* < 0.001) and Wt mice (*p* < 0.001). AS, *APP/PS1* sedentary group; AE, *APP/PS1* exercise group; CS, C57BL/6J sedentary group; CE, C57BL/6J exercise group. ***, *p* < 0.001.

The CA1 region of hippocampal slices derived from both Tg and Wt mice were utilized for recording fEPSPs. Following a stable 20-min baseline recording of fEPSPs, TBS was employed to evoke LTP, which was designed to mimic the firing patterns of hippocampal neurons during exploratory activity in vivo. As depicted in Figure 4, a two-way ANOVA analysis unveiled distinct influences of genotype (F_(1,39)_ = 54.282, *p* < 0.001) and exercise (F_(1,39)_ = 33.244, *p* < 0.001) on TBS-induced LTP within the hippocampus of 6-month-old Tg mice, without any notable interaction between these factors (F_(1,39)_ = 0.0233, *p* = 0.088). Notably, TBS triggered robust LTP in hippocampal slices from Wt mice, which remained stable throughout the 60-min recording session. Conversely, in Tg slices, the TBS-induced LTP gradually declined towards baseline levels. The slope of fEPSP, measured between 50 and 60 min post-TBS stimulation, was significantly attenuated in Tg mice compared to Wt mice (*p* < 0.001). However, an eight-week treadmill exercise regimen significantly enhanced the slope of fEPSP in both 6-month-old Tg (*p* < 0.001) and Wt mice (*p* < 0.001).

### 3.4. Treadmill Exercise Ameliorated Synaptic Pathology of the Hippocampus in 6-Month-Old Tg Mice

**Figure 5:**
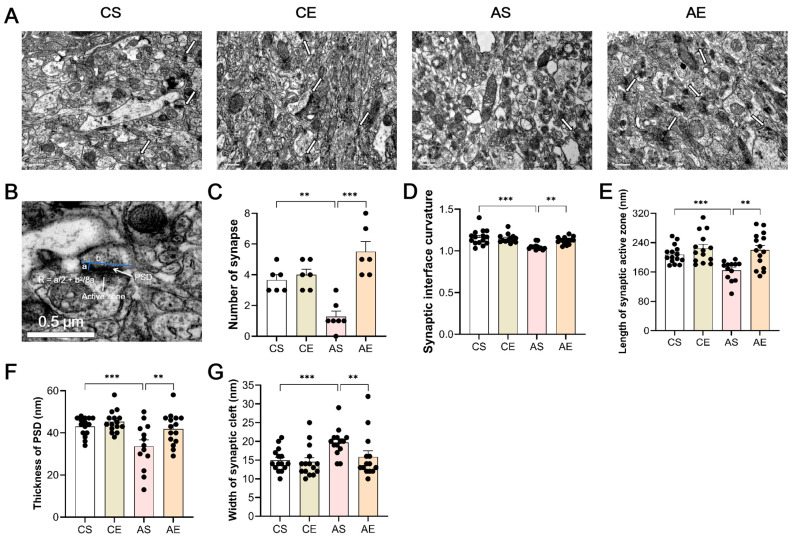
Treadmill exercise ameliorated synaptic pathology of the hippocampus in 6-month-old Tg mice. (**A**) Representative transmission electron microscope photos of different groups. (**B**) Representative measurement of synaptic structural parameters. When comparing the Tg mice to the CS group, notable deficiencies were observed in synapse number (**C**), *p* = 0.001), synaptic interface curvature ((**D**), *p* < 0.001), synaptic active zone length ((**E**), *p* = 0.003), and thickness of PSD ((**F**), *p* = 0.001), accompanied by a widened synaptic cleft ((**G**), *p* = 0.005). Remarkably, an eight-week treadmill regimen reversed these trends, significantly boosting synapse count ((**C**), *p* < 0.001), synaptic interface curvature ((**D**), *p* = 0.002), synaptic active zone number ((**E**), *p* < 0.001), and thickness of PSD ((**F**), *p* = 0.004) and narrowing the synaptic cleft ((**G**), *p* = 0.023) in Tg mice aged 6 months. Scale bar = 0.5 μm. AS, *APP/PS1* sedentary group; AE, *APP/PS1* exercise group; CS, C57BL/6J sedentary group; CE, C57BL/6J exercise group. **, *p* < 0.01; ***, *p* < 0.001.

Synaptic loss is a pivotal neuropathologic hallmark of AD [44]. We next determined the numbers of hippocampal synapses. As shown in Figure 5A,C, while genotype alone did not significantly impact synapse numbers (F_(1,24)_ = 0.965, *p* = 0.337), exercise exhibited a pronounced effect (F_(1,24)_ = 25.724, *p* < 0.001), with a notable interaction between genotype and exercise (F_(1,24)_ = 18.735, *p* < 0.001). Post hoc analysis revealed a significant reduction in hippocampal synapse numbers in Tg mice compared to the controls (*p* = 0.001), which was reversed by eight weeks of treadmill exercise (*p* < 0.001). In addition, compared with CE, the synapse numbers of the hippocampus in AE were significantly increased (*p* = 0.030).

To evaluate synaptic transmission efficiency, we analyzed several structural parameters. Regarding synaptic interface curvature (Figure 5D), genotype significantly influenced this metric (F_(1,55)_ = 17.412, *p* < 0.001), with exercise showing a trend towards significance (F_(1,55)_ = 3.554, *p* = 0.065) and a significant interaction between the two factors (F_(1,55)_ = 7.305, *p* = 0.009). Post hoc analysis revealed that Tg mice exhibited reduced curvature compared to the controls (*p* < 0.001), which was reversed by exercise (*p* = 0.002).

The length of the synaptic active zone (Figure 5E) was also affected by both genotype (F_(1,56)_ = 6.161, *p* = 0.016) and exercise (F_(1,56)_ = 14.099, *p* < 0.001), with a significant interaction (F_(1,56)_ = 4.115, *p* = 0.048). Post hoc analysis revealed that Tg mice had shorter active zones than the controls (*p* = 0.003), but exercise significantly elongated them (*p* < 0.001).

The thickness of the PSD (Figure 5F) was similarly influenced by genotype (F_(1,57)_ = 11.172, *p* = 0.002) and exercise (F_(1,57)_ = 7.389, *p* = 0.009), without a significant interaction (F_(1,57)_ = 2.47, *p* = 0.121). Post hoc analysis revealed that Tg mice displayed thinner PSDs compared to those of the controls (*p* = 0.001), which thickened significantly after exercise (*p* = 0.004).

Lastly, the width of the synaptic cleft (Figure 5G) was impacted by genotype (F_(1,57)_ = 7.121, *p* = 0.010) but not significantly by exercise (F_(1,57)_ = 3.463, *p* = 0.068), with no interaction observed (F_(1,57)_ = 2.284, *p* = 0.137). Post hoc analysis revealed that Tg mice had wider clefts than those of the controls (*p* = 0.005), which narrowed after exercise (*p* = 0.023), indicating improved synaptic organization.

### 3.5. Treadmill Exercise Increased the Expression and Activity of AMPAR in 6-Month-Old Tg Mice

**Figure 6:**
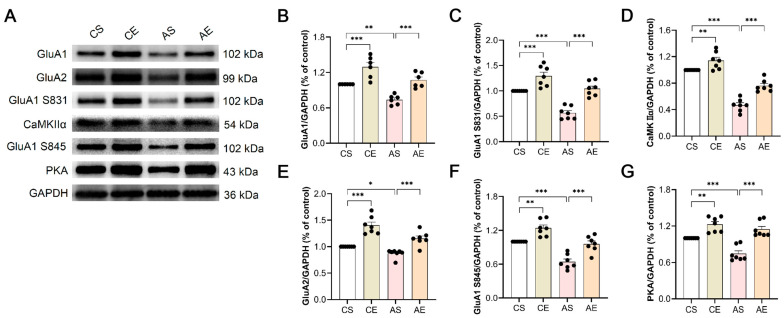
Treadmill exercise increased the expression and activity of AMPAR in 6-month-old Tg mice. (**A**) Representative immunoreactive bands detected by Western blot. In comparison to CS, the expression of GluA1 ((**B**), *p* = 0.001), GluA1 S831 ((**C**), *p* < 0.001), CaMKIIα ((**D**), *p* < 0.001), GluA2 ((**E**), *p* = 0.044), GluA1 S845 ((**F**), *p* < 0.001), and PKA ((**G**), *p* < 0.001) in the hippocampus of Tg mice is significantly diminished. Notably, an eight-week treadmill exercise regimen significantly elevates the expression of these proteins, including GluA1 ((**B**), *p* < 0.001), GluA1 S831 ((**C**), *p* < 0.001), CaMKIIα ((**D**), Tg: *p* < 0.001; Wt: *p* = 0.009), GluA2 ((**E**), *p* < 0.001), GluA1 S845 ((**F**), Tg: *p* < 0.001; Wt: *p* = 0.001), and PKA ((**G**), Tg: *p* < 0.001; Wt: *p* = 0.001) in both Tg and Wt mice. AS, *APP/PS1* sedentary group; AE, *APP/PS1* exercise group; CS, C57BL/6J sedentary group; CE, C57BL/6J exercise group. *, *p* < 0.05; **, *p* < 0.01; ***, *p* < 0.001.

Furthermore, we investigated the molecular changes in the different groups. The expression and activity of AMPARs were detected using Western blot. For GluA1 (Figure 6A,B), genotype (F_(1,23)_ = 24.457, *p* < 0.001) and exercise (F_(1,23)_ = 40.928, *p* < 0.001) significantly influenced its expression, with no interactive effect between the two (F_(1,23)_ = 0.175, *p* = 0.680). Tukey’s tests revealed a marked reduction in GluA1 expression in 6-month-old Tg mice compared to that in CS (*p* = 0.001), which was significantly reversed by 8 weeks of treadmill exercise in both Tg (*p* < 0.001) and Wt mice (*p* < 0.001).

Similar trends were observed for GluA1 S831 (Figure 6A,C), with genotype (F_(1,27)_ = 42.812, *p* < 0.001) and exercise (F_(1,27)_ = 54.567, *p* < 0.001) as significant factors, sans a significant interaction (F_(1,27)_ = 2.985, *p* = 0.097). Tukey’s analysis mirrored the GluA1 findings, with a decreased expression in Tg mice (*p* < 0.001) that was substantially upregulated by exercise in both Tg (*p* < 0.001) and Wt mice (*p* < 0.001).

In contrast, CaMKIIα exhibited a unique interaction between genotype and exercise (F_(1,27)_ = 4.746, *p* = 0.039), alongside significant main effects of genotype (F_(1,27)_ = 170.157, *p* < 0.001) and exercise (F_(1,27)_ = 38.465, *p* < 0.001). Tukey’s tests indicated a significant decrease in CaMKIIα expression in Tg mice (*p* < 0.001, Figure 6A,D), which was significantly enhanced by exercise in Tg (*p* < 0.001) and Wt mice (*p* = 0.009).

GluA2′s expression pattern mirrored that of GluA1 and GluA1 S831, with significant effects of genotype (F_(1,27)_ = 19.394, *p* < 0.001) and exercise (F_(1,27)_ = 63.414, *p* < 0.001) yet no interaction (F_(1,27)_ = 1.968, *p* = 0.173). Tukey’s analysis showed a decrease in GluA2 expression in Tg mice (*p* = 0.044, Figure 6A,E), which was significantly reversed by exercise in both Tg (*p* < 0.001) and Wt mice (*p* < 0.001).

For GluA1 S845 (Figure 6A,F), similar patterns emerged, with genotype (F_(1,27)_ = 50.709, *p* < 0.001) and exercise (F_(1,27)_ = 38.266, *p* < 0.001) effects prominent and no significant interaction (F_(1,27)_ = 0.689, *p* = 0.415). Tukey’s analysis demonstrated decreased GluA1 S845 expression in Tg mice (*p* < 0.001), which was significantly increased by exercise in Tg (*p* < 0.001) and Wt mice (*p* = 0.001).

Lastly, Figure 6A,G reveals that both genotype (F_(1,27)_ = 15.990, *p* < 0.001) and exercise (F_(1,27)_ = 56.700, *p* < 0.001) significantly modulated PKA expression, with a significant interaction between these variables (F_(1,27)_ = 4.685, *p* = 0.041). Similarly, Tg mice exhibited a notable decrease in PKA expression compared to that of CS (*p* < 0.001), which was significantly elevated following 8 weeks of treadmill exercise in both Tg (*p* < 0.001) and Wt mice (*p* = 0.001).

### 3.6. Treadmill Exercise Increased AMPAR/NMDAR of the Hippocampal Pyramidal Neurons in 6-Month-Old Tg Mice

**Figure 7:**
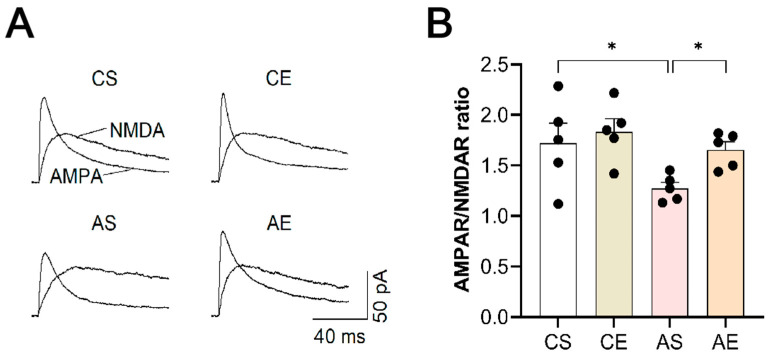
Treadmill exercise increased ANR of the hippocampal pyramidal neurons in 6-month-old Tg mice. (**A**) Representative EPSCs recorded from hippocampal pyramidal neurons in slices prepared from different groups. (**B**) The decrease in ANR observed in Tg mice was reversed by treadmill exercise (*p* = 0.048). AS, *APP/PS1* sedentary group; AE, *APP/PS1* exercise group; CS, C57BL/6J sedentary group; CE, C57BL/6J exercise group. *, *p* < 0.05.

ANR has been used as an indicator of synaptic potentiation in vivo, measuring the relative expression of AMPAR and NMDAR in synapses. As depicted in Figure 7, a two-way ANOVA analysis uncovered a significant effect of genotype (F_(1,19)_ = 6.168, *p* = 0.024) on ANR, whereas exercise did not exhibit a notable influence (F_(1,19)_ = 3.777, *p* = 0.070), and no interaction was observed between these factors (F_(1,19)_ = 1.129, *p* = 0.304). Tukey’s analysis further revealed that, in comparison to that of CS, the ANR of hippocampal pyramidal neurons in 6-month-old Tg mice was significantly reduced (*p* = 0.023), which was significantly increased following 8 weeks of treadmill exercise (*p* = 0.048).

## 4. Discussion

### 4.1. Main Findings

In this study, we examined the influence of treadmill exercise on behavioral performance and associated molecular mechanisms, employing 6-month-old *APP/PS1* mice that exhibit an age-progressive neurological decline resembling AD symptoms [45]. We demonstrated that treadmill exercise positively impacted memory retrieval, bolstering basal hippocampal synaptic strength, synaptic plasticity, and synaptic morphology. Additionally, it augmented the expression of GluA1, GluA2, GluA1 S831, GluA1 S845, and their phosphorylation-linked proteins in 6-month-old *APP/PS1* mice.

### 4.2. Behavioral Performance and Synaptic Plasticity

Exercise, a ubiquitous habit, triggers cellular and molecular reactions essential for sustaining and enhancing brain plasticity. Recent studies underscore the cognitive benefits of exercise for AD patients [26,46]. Notably, we initiated treadmill exercise (50–65% maximal oxygen uptake) at 4 months of age, preceding the typical emergence of Aβ deposits around 6 months [37]. Our observations concurred with prior reports [45,47], demonstrating memory decline in 6-month-old *APP/PS1* mice. Remarkably, an 8-week treadmill exercise regimen reversed memory deficits in these mice, aligning with studies that precede Aβ accumulation with intervention [10,48]. This, coupled with findings from treadmill exercise in other AD models [28,29,49], indicates its potential as an early intervention to delay AD progression.

The I/O curve serves as a crucial metric for assessing basic synaptic strength and synaptic transmission in the hippocampus. Its shape reflects the number of fibers activated by varying stimulus intensities, as manifested in the compound action potential (CAP). Variations in synaptic strength alter the I/O curve’s position. An enhancement in synaptic strength would shift the curve leftward, signifying an increased CAP amplitude at a given stimulus intensity [50]. The present results showed a rightward shift and reduced AMPAR-mediated fEPSPs, indicating weakened basal synaptic strength in 6-month-old *APP/PS1* mice. However, 8 weeks of treadmill exercise significantly elevated AMPAR-mediated fEPSPs in both *APP/PS1* and C57BL/6J mice, pointing to its efficacy in strengthening hippocampal synaptic function.

PPF, a presynaptic Ca^2+^-dependent STP phenomenon, mirrors the probability of neurotransmitter vesicle release due to a short-term enhancement in synaptic transmission efficiency when presynaptic neurons are stimulated with two consecutive short-interval stimuli [51]. Alternations in PPF indicate changes in the presynaptic release probability. The results showed that PPF at 20–400 ms interpulse intervals was significantly decreased in 6-month-old *APP/PS1* mice, whereas 8 weeks of treadmill exercise significantly reduced PPF in both *APP/PS1* and C57BL/6J mice, suggesting an enhancement in the presynaptic release probability within the hippocampus of these mice.

Moreover, hippocampal LTP, a pivotal synaptic plasticity process linked to learning and memory, has garnered attention in elucidating cognitive decline mechanisms associated with AD [52]. To delve deeper into the electrophysiological underpinnings of treadmill exercise’s behavioral benefits, we conducted LTP recordings. In 6-month-old 57BL/6J mice, TBS robustly induced LTP that remained stable over a 60-min period. Conversely, in age-matched *APP/PS1* mice, TBS-elicited LTP gradually declined towards baseline levels, with a significantly reduced slope between 50 and 60 min post-stimulation, aligning with prior findings of hippocampal LTP suppression in these mice [9,53,54]. Intriguingly, 8 weeks of treadmill exercise significantly augmented LTP in both 6-month-old *APP/PS1* and Wt mice, echoing previous studies demonstrating exercise’s potential to bolster hippocampal LTP in this model [10,49,55]. Given the intimate connection between cognitive function and LTP, the exercise-induced LTP facilitation represents a cellular mechanism that may underlie the behavioral improvements observed in 6-month-old *APP/PS1* mice following treadmill exercise.

### 4.3. Synaptic Pathology

The efficacy of learning and memory processes is intricately tied to the abundance and functionality of synapses, the fundamental units of neuronal communication within the brain. As mentioned above, the typical onset of Aβ deposition is usually around 6 months [37], and several studies have shown that synaptic loss is at least partially related to Aβ-mediated toxicity [56,57,58]. Beyond the accumulation of pathological hallmarks like plaques and tangles, synaptic loss stands as a defining feature of AD, with a robust correlation between cognitive decline and synapse depletion observed [59,60]. Epidemiological evidence further underscores the causative link between reduced synaptic integrity and cognitive impairments in AD [61]. Our results revealed a marked decrease in hippocampal synapse numbers at the inception of Aβ deposition in 6-month-old *APP/PS1* mice. Notably, an 8-week treadmill exercise regimen significantly reversed this trend, augmenting synapse numbers in line with the observations made by Li et al. [62].

Structural plasticity in the hippocampus manifests through alterations in synaptic architecture, encompassing changes in the synapse number, synaptic interface curvature, length of the synaptic active zone, thickness of the PSD, and width of the synaptic cleft [63]. This intricate interplay forms the cellular cornerstone of learning and memory processes [64,65]. Specifically, the length of the synaptic active zone mirrors presynaptic mechanisms, while postsynaptic dynamics are reflected in the synaptic interface curvature and thickness of the PSD [66,67]. The synaptic cleft, as the conduit for neurotransmitter transfer, represents a critical structural component [68]. Modifications to synaptic ultrastructure can subsequently impact synaptic function, underscoring its role as a structural basis for synaptic plasticity [69].

Our results revealed significant alterations in the hippocampal synaptic architecture of 6-month-old *APP/PS1* mice, marked by a reduction in the synaptic interface curvature, length of the synaptic active zone, and thickness of the PSD, alongside an expansion of the synaptic cleft width. Conversely, treadmill exercise promoted favorable morphological adjustments in these mice, characterized by enhancements in the aforementioned parameters and a narrowing of the synaptic cleft, echoing the findings of Li et al. [62]. Furthermore, the elongation of the synaptic active zone concurred with the PPF results. This positive impact extended to behavioral domains, as evidenced by improvements in memory retrieval, basic synaptic strength, STP, and LTP, all of which aligned with the observed synaptic structural amelioration. In addition, the results of the length of the synaptic active zone were consistent with those of PPF, suggesting that treadmill exercise improved the hippocampal synaptic plasticity in 6-month-old *APP/PS1* mice through presynaptic neuronal mechanisms. Collectively, these results underscore the efficacy of treadmill exercise in reversing pathological changes in synapse numbers and the synaptic ultrastructure, fostering a more favorable synaptic morphology in 6-month-old *APP/PS1* mice. This synaptic structural optimization serves as the underlying mechanism through which exercise enhances synaptic plasticity and behavioral outcomes, demonstrating its therapeutic potential.

### 4.4. Expression and Activity of AMPAR

LTP is induced by specific patterns of neuronal activity. For LTP induction, both pre- and postsynaptic neurons need to be active simultaneously, as the postsynaptic neuron must be depolarized when glutamate is released from the presynaptic axonal terminals. This depolarization fully relieves the Mg^2+^ block of NMDARs. The coincident depolarization and glutamate binding maximizes calcium influx through NMDARs, which activates intracellular signaling cascades responsible for the changes in synaptic efficacy [70]. Thus, changed NMDAR activation may contribute to enhanced LTP. However, Megill et al. [71] demonstrated that the LTP deficit observed in 6-month-old *APP/PS1* mice was not due to alterations in induction mechanisms. Additionally, they showed that the expression levels of NMDAR subunits GluN2A (NR2A) and GluN2B (NR2B) remained unchanged, suggesting that LTP induction mechanisms were unaffected. Therefore, we focused on AMPARs but not NMDARs in the present study.

The primary mediator of rapid synaptic transmission, AMPAR, undergoes trafficking alterations that are pivotal to synaptic plasticity [72]. A recent study noted a notable decline in GluA1 expression in 4-month-old *APP/PS1* mice [73], emphasizing the significance of GluA1 phosphorylation in LTP, as evidenced by multiple studies [74]. During LTP induction, GluA1 C-tail residue S831 is phosphorylated by PKC and CaMKII, which affects AMPAR trafficking and the single-channel conductance of AMPAR. In addition, GluA1 C-tail residue S845 can be phosphorylated by PKA, which leads to increased trafficking and insertion of AMPAR at dendritic spine synapses [75]. Our study delved into the expression of AMPAR subunits GluA1 and GluA2, along with GluA1′s phosphorylated forms at S831 and S845, and the associated kinases CaMKIIα and PKA in the hippocampus of 6-month-old *APP/PS1* mice. Notably, we observed a significant reduction in the expression of these proteins and their phosphorylated forms. However, an 8-week treadmill exercise regimen significantly elevated their expression levels in both 6-month-old *APP/PS1* and C57BL/6J mice, corroborating previous findings [62,76,77].

These results were consistent with the results of synaptic interface curvature and thickness of the PSD, suggesting that treadmill exercise improved the synaptic plasticity through postsynaptic neuronal mechanisms. Previous work with GluA1 knockout mice underscores GluA1′s indispensability for hippocampal LTP and spatial memory [78]. Furthermore, phosphorylation of GluA1 at S831 and S845 has been shown to facilitate LTP induction, thereby augmenting synaptic plasticity [79]. Collectively, our findings highlight the therapeutic potential of treadmill exercise in modulating AMPAR-mediated synaptic plasticity and associated cognitive functions.

### 4.5. AMPAR/NMDAR Ratio

Finally, we determined whether changes in AMPARs were caused by AMPA trafficking from the plasma membrane and therefore examined the ANR, an indicator of synaptic strength in hippocampal pyramidal neurons, which measures the relative expression of AMPARs and NMDARs at the synapse. As we had predicted, 6-month-old *APP/PS1* mice exhibited a pronounced reduction in ANR, suggesting an attenuation of excitatory synaptic transmission strength. However, 8 weeks of treadmill exercise significantly reversed this attenuation and increased ANR. 

Taken together, treadmill exercise-induced upregulation of hippocampal AMPARs expression and activity was consistent with improved LTP in 6-month-old *APP/PS1* mice. This molecular-level adjustment forms the cornerstone for the exercise-mediated improvements in synaptic plasticity and associated behavioral outcomes in these mice.

## 5. Conclusions

In this study, we demonstrated that treadmill exercise facilitated synaptic plasticity by increasing the structural synaptic plasticity of the hippocampus as well as the activity of AMPARs in 6-month-old *APP/PS1* mice. To the best of our knowledge, this is the first study demonstrating that exercise facilitated STP and LTP of the hippocampus, which highlights the electrophysiological mechanisms underlying the improved behavioral performance in 6-month-old *APP/PS1* mice.

## Data Availability

The datasets used and/or analyzed during the current study are available from the corresponding author on reasonable request.
